# Case Report: Total intragastric mesh migration six years after diaphragmatic rupture and hiatal hernia surgery

**DOI:** 10.12688/f1000research.139090.1

**Published:** 2023-07-24

**Authors:** Asma Sghaier, Mohamed Amine Elghali, Abdelrahmen Daadoucha, Amal Letaief, Itimed GHARBI, Fehmi Hamila, Sabri Youssef

**Affiliations:** 1 Surgery, Hospital Farhat Hached of Sousse, Sousse, Tunisia; 2 General Surgery, Faculty of Medicine of Sousse, Sousse, Tunisia; 3 Radiology, Hospital Ibn Al Jazzar Kairouan, Kairouan, Tunisia; 4 Radiology, Faculty of Medicine of Sousse-University of Sousse, Sousse, Tunisia

**Keywords:** diaphragmatic rupture, hiatal hernia, mesh repair, complications

## Abstract

**Background:** Mesh implementation to repair the hiatal space is already justified. Nevertheless, the use of this procedure is debated in regard of complications that may occur. Mesh erosion and migration are considered the most serious complications of mesh repairs.

**Case presentation:** It has not yet been well described in the literature. We describe a case of mesh erosion of stomach, many years later after a prosthetic repair of a diaphragmatic rupture associated to hiatal hernia, is presented here because of its rarity.

**Conclusion:** Few explanations have been put forward to explain this incident. Could it be due to inflammatory processes, or to the composition of the Meshes? As yet, there is no definitive explanation.

## Introduction

Delayed presentation of traumatic diaphragmatic rupture is a challenging diagnostic and treatment with necessity of mesh reinforcement. ^1^ Mesh repair can also be proposed for hiatal hernia. ^2^ There are a few published observational studies supporting the use of mesh, convincing lower rates of recurrence with lack of long-term follow-up. Likewise, there are sparse published studies proving the complications associated with mesh in the long-term, ^3^ which provided the importance for this case. Limited related complications of mesh in such localization have been described. We describe one case of late dysphagia due to intragastric mesh migration six year after surgical reparation for diaphragmatic rupture associated with hiatal hernia.

## Case report

**Figure 1.**
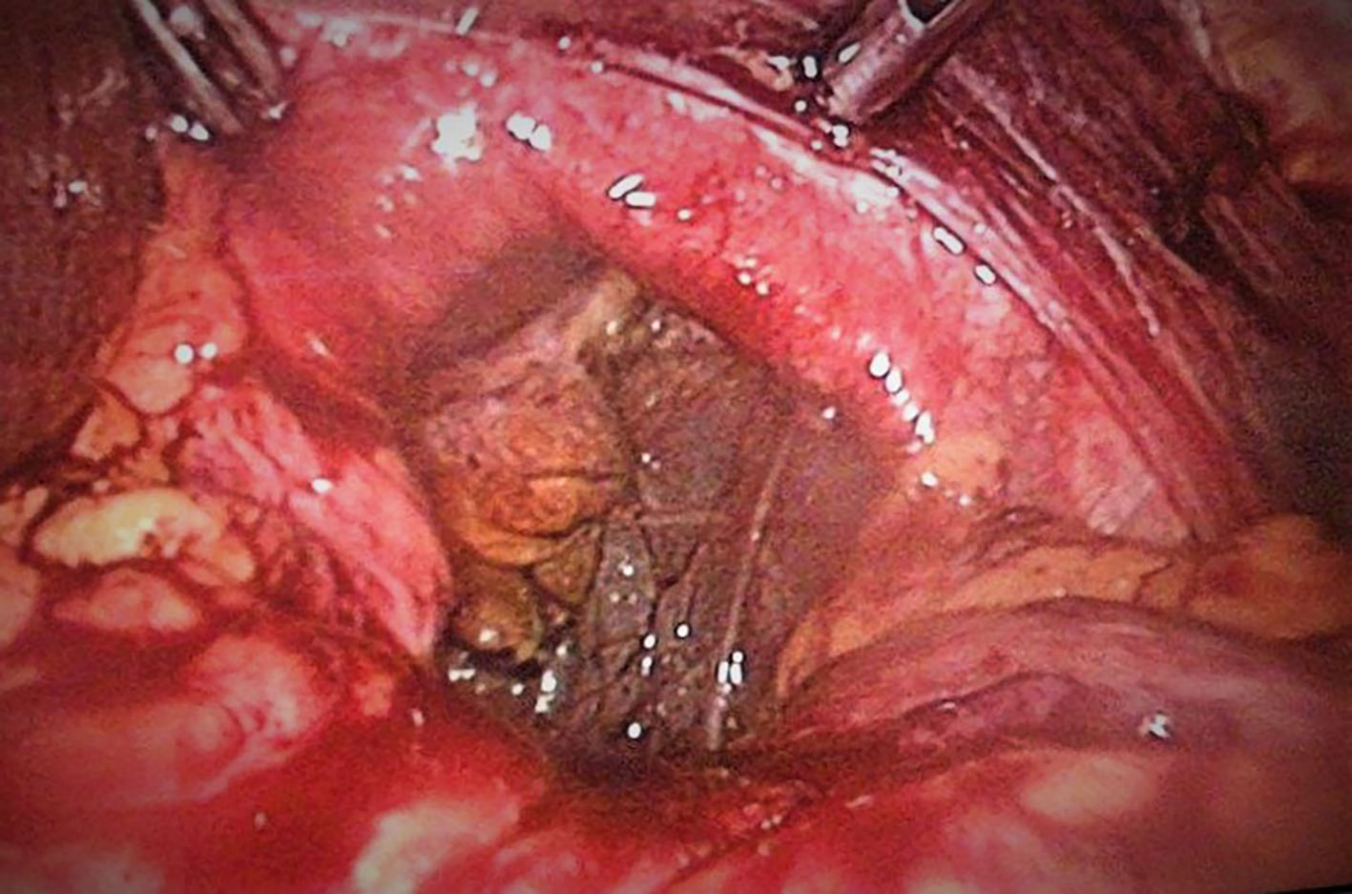
Diaphragmatic defect.

A 52-year-old white man, with history of epigastric pain and gastroesophageal reflux disease, was operated for mixt hiatal hernia (Type III). During the operation we found that it was a left diaphragmatic rupture with a large collar with gastric migration into the thorax. The diaphragmatic hernia is located 5 cm from a wide hiatus with a sliding hiatal hernia ( Figure 1).

**Figure 2.**
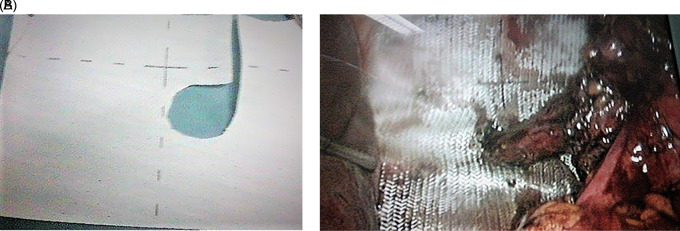
A: Plaque double face. B: Intraoperative view of mesh placement.

Intraperitoneal gastric reduction and Nissen fundoplication was performed. A two-face mesh 15×15 cm was put in place covering the orifice of the diaphragmatic rupture and the hiatal orifice by tying the esophagus ( Figure 2). Fixation was performed by crown tacks. Post-operative course was simple. The patient required level I analgesics. Transit was restored after 24 hours. The patient described no complaints, particularly no dysphagia, and the physical examination was normal.

By interviewing the patient post-operatively, we discovered that the patient, who belongs to the forces of order. He was a security officer and he was a victim of an accident during a pursuit and was violently struck with the wheel of his car. He consulted as a matter of emergency and had an abdominal ultrasound and was put out with analgesic treatment.

**Figure 3.**
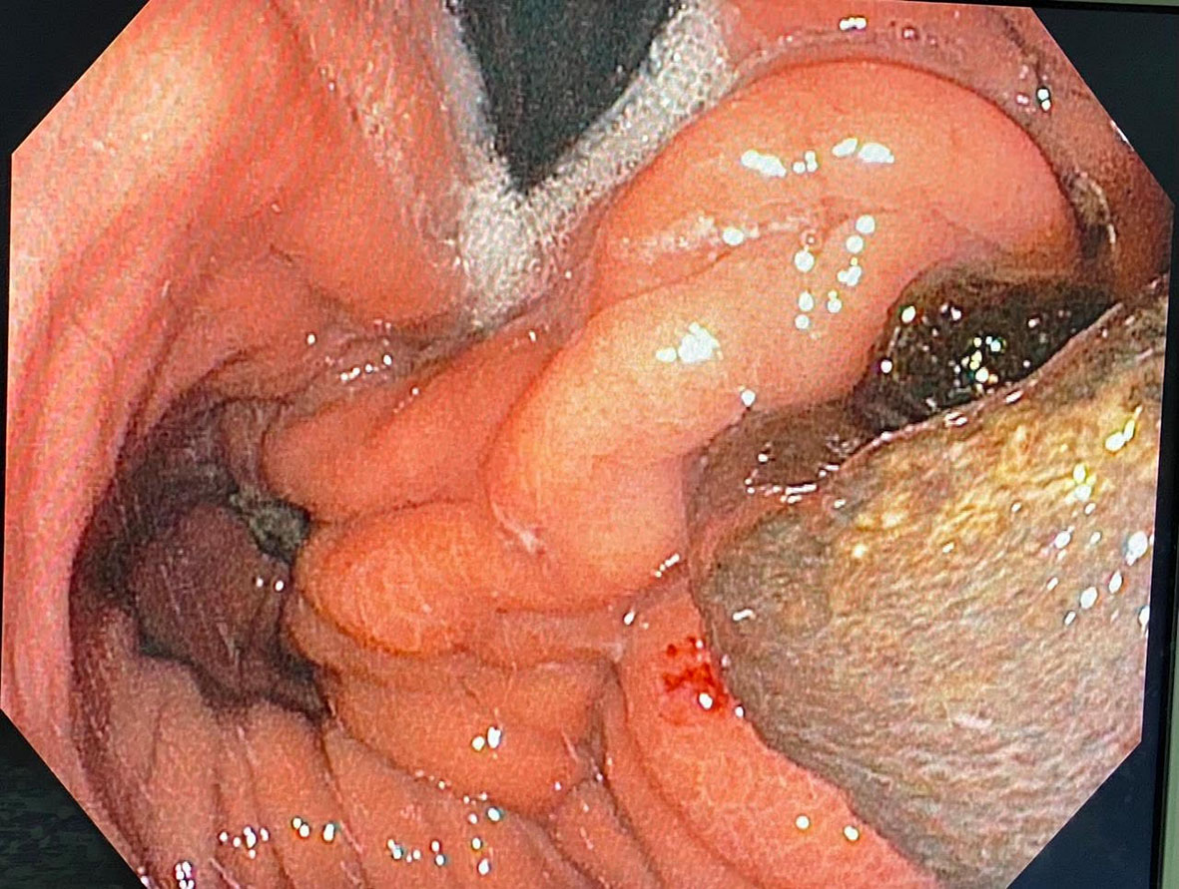
Endoscopic view of the mesh into stomach.

The patient remained asymptomatic for more than six years postoperatively. When, he had progressive complaints of dysphagia and weight loss. An upper gastro-intestinal endoscopy showed a dilated esophagus, crossed cardia easily without protrusion with evidence of anti-reflux montage and migration of mesh into the stomach. Pneumatic dilation of antireflux montage with a 30-mm balloon was performed ( Figure 3).

An attempt at endoscopic removal of the mesh had failed. A magnetic resonance imaging examination had not described any signs in favor of a diaphragmatic defect.

The decision was then made to operate the patient laparoscopically and to remove the mesh.

**Figure 4.**
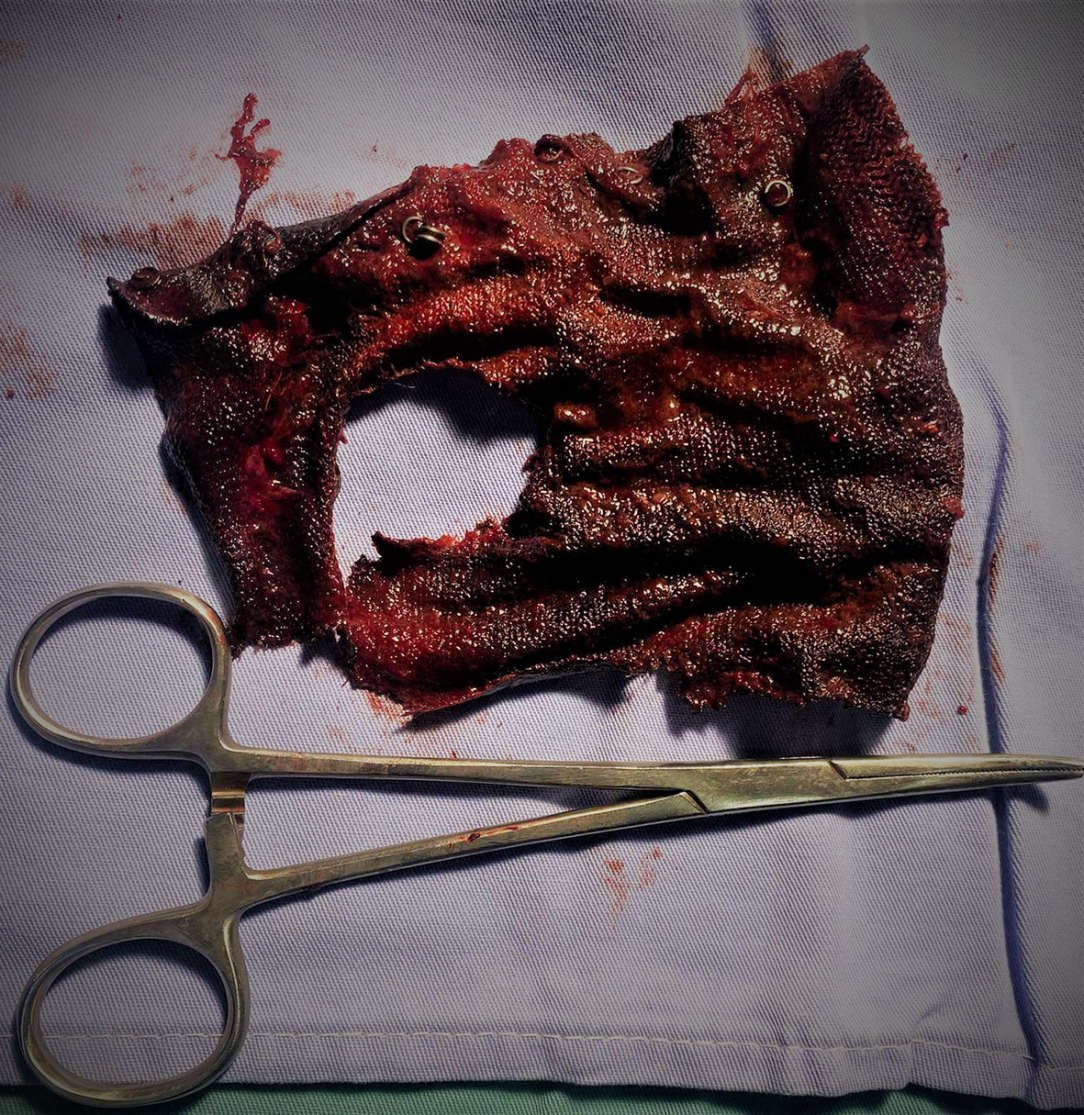
Mesh after surgical extraction.

The patient was operated on, the per operative exploration discovered the presence of several adhesions at the supra meso-colic level. A longitudinal gastrotomy was carried out which made it possible to reveal the mesh that was inside and to extract it with the few staples that were attached ( Figure 4).

The postoperative course was simple. The patient was discharged at day six.

## Discussion

Mesh repair is becoming increasingly necessary to manage large diaphragmatic rupture. ^4^ But use of mesh at the hiatus is yet controversial because of possible complications that may occur. ^5^^–^^7^ Recommendation with a fairly high level of evidence concerning indications of prosthetic mesh does not clearly developed. Many procedures were proposed. Tension-free procedure consist of mesh setting without tight suture to procure excessive tension whereas the on-lay technique with consolidation of the defect closure by mesh. The materiel could be placed in an anterior, posterior, or circular position with a hole for the passage of the esophagus. ^7^

The mesh used for hiatal reinforcement are made usually of non-resorbable material. It should have a very low risk of post-operative adhesions, and can be easy to be manipulate proceeding by laparoscopic approach.

Polypropylene mesh seems to offer most of these requirements; however, it is susceptible to be responsible of intraperitoneal adhesions and also sometimes fistulas. ^8^ Prosthetic reinforcement, though associated with a low rate of hernia recurrence, has particular drawbacks like this case of migration into stomach that we described in this article. Indeed, there are two considerable complications due to meshes: Parietal erosion and esophageal stenosis. The incidences of these two main complications ranged in literature between 0%–0.49% ^8^ and 3.9% ^9^ respectively depending on the series. However, surgical management is crucial to treat both conditions. A predisposition to esophageal stenosis succeeding the setting up of meshes produced by biological materials and toward parietal erosion after the use of polytetrafluoroethylene and polypropylene meshes has been developed by retrospective studies. ^10^ Furthermore, stitches around a mesh placed above the fundoplication may be responsible of dysphagia, and contact of the mesh with the esophagus may leads to erosion, as illustrated in our case. Moreover, a surgical approach might provide a crucial role in decreasing the incidence of complications due to mesh. ^10^ The techniques proposed for this purpose are tension-free repair with mesh placement without crus suturing to avoid excessive tension and the on-lay technique with reinforcement of the crucial closure by mesh. The on-lay technique is usually performed in all cases of hiatal hernia, independently of hernia size. ^11^

Further recent studies concluded that mesh might be associated with fewer short-term recurrences, and the biological mesh was involved with improved short-term quality of life. Nevertheless, these advantages were offset by more dysphagia, ^12^ which is why most experienced practitioners recommend mesh use exclusively for carefully selected cases. ^13^ For our patient we could not explain this intragastric migration of the Mesh. Would it be related to the fixation by the tackers and the nature of the Mesh? We do not have valid proof and explanations.

## Conclusions

Our case, even though it is rare, demonstrates this, as we are led to re-operate a patient and to be faced with technical difficulties and often even a conversion to open surgery to retrieve the mesh that migrated inside the digestive tract. The mesh type may provide a role in the complication rate, with synthetic mesh being more implicated. The simultaneous co-existence of an intraperitoneal infection may also be responsible, although that must be proven by well-conducted studies and further controlled randomized trials.

## Consent for publication

Written informed consent was obtained from the patient for publication of this case report and accompanying images.

## Data Availability

No data are associated with this article.
