# Supplementary material for: Case Report: Total intragastric mesh migration six years after diaphragmatic rupture and hiatal hernia surgery
Source: F1000Res. 2025 Mar 7;12:871. Originally published 2023 Jul 24. [Version 2] doi: 10.12688/f1000research.139090.2 (PMC11904395; doi:10.12688/f1000research.139090.2)
Supplement: Peer review history

# Reviewer report: Reviewer response for version 2

**DOI:** 10.5256/f1000research.178358.r369923

**Published:** 2025-03-12

**Authors:** Nir Messer

**Affiliations:** 1 Cleveland Clinic Foundation, Cleveland, USA

I have read the authors' responses and reviewed the updated version of the manuscript.

 The authors have made significant revisions in response to the feedback provided, and although there are still some imperfections, the manuscript has improved substantially. I believe that it now meets the requirements of the journal and is suitable for acceptance.

Are enough details provided of any physical examination and diagnostic tests, treatment given and outcomes?

Partly

Is the case presented with sufficient detail to be useful for other practitioners?

No

Is sufficient discussion included of the importance of the findings and their relevance to future understanding of disease processes, diagnosis or treatment?

No

Is the background of the case’s history and progression described in sufficient detail?

No

Reviewer Expertise:

Abdominal Wall reconstruction, paraesophageal hernia repair, endocrine surgery, and general surgery.

I confirm that I have read this submission and believe that I have an appropriate level of expertise to confirm that it is of an acceptable scientific standard.

# Reviewer report: Reviewer response for version 1

**DOI:** 10.5256/f1000research.152335.r234856

**Published:** 2024-01-29

**Authors:** Nir Messer

**Affiliations:** 1 Cleveland Clinic Foundation, Cleveland, USA

**Total intragastric mesh migration six years after diaphragmatic rupture and hiatal hernia surgery**

 I value the opportunity to assess the study "Total intragastric mesh migration six years after diaphragmatic rupture and hiatal hernia surgery". The manuscript presents a case study involving mesh migration following diaphragmatic rupture and hiatal hernia repair with permanent mesh. I find this case to be interesting and challenging, and I would like to provide the following comments:

**Case presentation**

1. " A 52-year-old white man, with history of epigastric pain and gastroesophageal reflux disease, was operated for mixt hiatal hernia (Type III)". The presentation is confusing. The authors initially label the patient with type III paraesophageal hernia, yet later, it is characterized as a sliding hiatal hernia (type I). Kindly provide an accurate clarification regarding the suspected and confirmed diagnosis for the patient.
2. Please specify the preoperative diagnostic procedures employed for the patient.
3. "Intraperitoneal gastric reduction and Nissen fundoplication were performed". Was there concurrent hiatal hernia repair involving hernia sac reduction and crural reapproximation?
4. "A two-face mesh 15×15 cm was put". Kindly specify the precise type of mesh utilized in the procedure.

**Discussion**

1. "Polypropylene mesh seems to offer most of these requirements; however, it is susceptible to be responsible of intraperitoneal adhesions and also sometimes fistulas". Polypropylene mesh is generally not recommended for intraabdominal application unless it is coated with an anti-adhesive material, attributed to its elevated adhesion and bowel erosion characteristics.
2. "The techniques proposed for this purpose are tension-free repair with mesh placement without crus suturing to avoid excessive tension and the on-lay technique with reinforcement of the crucial closure by mesh. The on-lay technique is usually performed in all cases of hiatal hernia, independently of hernia size". As highlighted in numerous studies, the predominant method for para-esophageal hernia repair involves crural reapproximation, even when incorporating mesh. The term "on-lay technique" is inaccurately applied in this context.

"whereas the on-lay technique with the consolidation of the defect closure by mesh". This sentence lacks clarity. Additionally, the term "on-lay" is typically employed to describe mesh positioning in abdominal wall hernias, not diaphragmatic hernias.

**General comments**

1. The manuscript lacks clarity, requiring repeated readings for comprehension. Furthermore, authors utilize inappropriate surgical jargon for diaphragmatic hernia repairs and attempt to extrapolate motives from abdominal wall hernia repairs to diaphragmatic hernias, a mismatch that warrants attention.
2. The figures lack clarity and fail to enhance the comprehension of the presented case.

Are enough details provided of any physical examination and diagnostic tests, treatment given and outcomes?

Partly

Is the case presented with sufficient detail to be useful for other practitioners?

No

Is sufficient discussion included of the importance of the findings and their relevance to future understanding of disease processes, diagnosis or treatment?

No

Is the background of the case’s history and progression described in sufficient detail?

No

Reviewer Expertise:

Abdominal Wall reconstruction, paraesophageal hernia repair, endocrine surgery, and general surgery.

I confirm that I have read this submission and believe that I have an appropriate level of expertise to state that I do not consider it to be of an acceptable scientific standard, for reasons outlined above.

# Response

**Published:** 2025-02-16

**Authors:** Asma SGHAIER

**Affiliations:** GENERAL SURGERY, FACULTY OF MEDICINE OF SOUSSE, SOUSSE, Tunisia

**Detailed Response to Reviewers**

**Submission date: 16/02/2025**

**Dear Professor Nir Messer**

 Thank you for your letter and for the reviewers’ comments concerning our manuscript.

 Thank you very much for your consideration, and we really appreciate the comments and have learned a lot. Appropriate changes were made according to the suggestions of reviewers and editor.

 Those comments are all valuable and very helpful for revising and improving our paper, as well as the important guiding significance to our researches. We have studied comments carefully and have made correction which we hope meet with approval.

 1-The investigations carried out preoperatively were in favour of a type III hernia, but intraoperatively it was more likely to be a type I hernia. Interpretations were performed by an expert medical staff. These lapses are to be expected and should not in any way be allowed to interfere with therapeutic management.

 2-The preoperative diagnosis was based on endoscopic and CT scan findings, which are not the aim of this report.

 3-reduction of the hernial sac and repair of the defect using the insertion of the folds of the diaphragm were performed.

 We have made the required changes to improve the quality of our manuscript.

 We would hope to provide you with a response to all your comments.

 We express once again our sincere appreciation and deep gratitude to all the reviewers and to the editorial board for providing us with the opportunity to revise our manuscript and to improve the quality of our submission.

 We hope, nevertheless, to receive positive feedback and acceptance of our work by allowing it to be published in this prestigious journal.

 Sincerely

 The corresponding author

# Reviewer report: Reviewer response for version 1

**DOI:** 10.5256/f1000research.152335.r205919

**Published:** 2023-10-10

**Authors:** Ivan Romic

**Affiliations:** 1 Department of Surgery, University Hospital Centre Zagreb, Zagreb, Croatia

Very interesting article on rare complication of hiatal hernia surgery. It provides details of diagnostic and management. 

- I suggest to state what kind of MESH was used, which material and company. 
- Also, reformulate 'belongs' to 'belonged'.
- "2. Our case, even though it is rare, demonstrates this, as we are led to re-operate a patient and to be faced with technical difficulties and often even a conversion to open surgery to retrieve the mesh that migrated inside the digestive tract." -  this is unclear, reformulate.
- Please find and cite all articles on MESH migration. One of them is attached ^1^.

Are enough details provided of any physical examination and diagnostic tests, treatment given and outcomes?

Yes

Is the case presented with sufficient detail to be useful for other practitioners?

Yes

Is sufficient discussion included of the importance of the findings and their relevance to future understanding of disease processes, diagnosis or treatment?

Yes

Is the background of the case’s history and progression described in sufficient detail?

Yes

Reviewer Expertise:

Abdominal surgery, gastric surgery

I confirm that I have read this submission and believe that I have an appropriate level of expertise to confirm that it is of an acceptable scientific standard.

## References

[1] : Endoscopic removal of migrated Nissen fundoplication mesh. *VideoGIE* .2020;5(6) : 10.1016/j.vgie.2020.02.010 238-240 10.1016/j.vgie.2020.02.010 32529156 PMC7277046

# Response

**Published:** 2025-02-16

**Authors:** Asma SGHAIER

**Affiliations:** GENERAL SURGERY, FACULTY OF MEDICINE OF SOUSSE, SOUSSE, Tunisia

**Detailed Response to Reviewers**

**Dear Professor** **Ivan Romic**

 Thank you for your letter and for the reviewers’ comments concerning our manuscript.

 Thank you very much for your consideration, and we really appreciate the comments and have learned a lot. Appropriate changes were made according to the suggestions of reviewers and editor.

 Those comments are all valuable and very helpful for revising and improving our paper, as well as the important guiding significance to our researches. We have studied comments carefully and have made correction which we hope meet with approval.

 The MESH used is a polypropylene material with two faces, one water- resistant and the other water-repellent. we are under a moral obligation to refrain from using the brand name for ethical reasons and to avoid any conflict of interest.

 There were several fibrous adhesions which were difficult to dissect. Attempts to access the supra-mesocolic floor and hiatal region were unsuccessful and not safe. Conversion to open surgery was mandatory.

 The necessary revisions were included in the manuscript

**We express once again our sincere appreciation and deep gratitude to all the reviewers and to the editorial board for providing us with the opportunity to revise our manuscript and to improve the quality of our submission.**

**We hope, nevertheless, to receive positive feedback and acceptance of our work by allowing it to be published in this prestigious journal.**

**Sincerely**

**The corresponding author**
